# WSN-Assisted UAV Trajectory Adjustment for Pesticide Drift Control

**DOI:** 10.3390/s20195473

**Published:** 2020-09-24

**Authors:** Jie Hu, Tuan Wang, Jiacheng Yang, Yubin Lan, Shilei Lv, Yali Zhang

**Affiliations:** 1 College of Electronic Engineering, South China Agricultural University, Wushan Road, Guangzhou 510642, China; hjgz79@scau.edu.cn (J.H.); twang@stu.scau.edu.cn (T.W.); yangjc@stu.scau.edu.cn (J.Y.); ylan@scau.edu.cn (Y.L.); lvshilei@scau.edu.cn (S.L.); 2 National Center for International Collaboration Research on Precision Agricultural Aviation Pesticide Spraying Technology, Wushan Road, Guangzhou 510642, China; 3 College of Engineering, South China Agricultural University, Wushan Road, Guangzhou 510642, China

**Keywords:** WSN, UAV, trajectory adjustment, drift control, DQN, PSO

## Abstract

Unmanned Aerial Vehicles (UAVs) have been widely applied for pesticide spraying as they have high efficiency and operational flexibility. However, the pesticide droplet drift caused by wind may decrease the pesticide spraying efficiency and pollute the environment. A precision spraying system based on an airborne meteorological monitoring platform on manned agricultural aircrafts is not adaptable for. So far, there is no better solution for controlling droplet drift outside the target area caused by wind, especially by wind gusts. In this regard, a UAV trajectory adjustment system based on Wireless Sensor Network (WSN) for pesticide drift control was proposed in this research. By collecting data from ground WSN, the UAV utilizes the wind speed and wind direction as inputs to autonomously adjust its trajectory for keeping droplet deposition in the target spraying area. Two optimized algorithms, namely deep reinforcement learning and particle swarm optimization, were applied to generate the newly modified flight route. At the same time, a simplified pesticide droplet drift model that includes wind speed and wind direction as parameters was developed and adopted to simulate and compute the drift distance of pesticide droplets. Moreover, an LSTM-based wind speed prediction model and a RNN-based wind direction prediction model were established, so as to address the problem of missing the latest wind data caused by communication latency or a lack of connection with the ground nodes. Finally, experiments were carried out to test the communication latency between UAV and ground WSN, and to evaluate the proposed scheme with embedded Raspberry Pi boards in UAV for feasibility verification. Results show that the WSN-assisted UAV trajectory adjustment system is capable of providing a better performance of on-target droplet deposition for real time pesticide spraying with UAV.

## 1. Introduction

Crop protection UAVs have achieved rapid development in recent years for their high pesticide spraying efficiency and strong environmental adaptation. Meanwhile, greater demands have also been made for precision pesticide spraying along with the advancement of precision agriculture. Pesticide droplet deposition is a critical indicator for measuring the effect of precision pesticide spraying. The wind speed and wind direction in meteorological conditions have greater influences on the deposition and drift of the pesticide droplets, in comparison to the flying height of the UAV and the droplet size, etc. In particular, the crosswind-induced pesticide drift, which can be defined as the process whereby the pesticide droplet disappears or re-settles after being carried out of the target area by the airflow perpendicular to its motion, is a principal factor affecting aerial spraying [1]. Since the pesticide droplet drifting outside of the target area could lead to the ineffective spraying dosage of pesticides, it not only affects the control efficiency and reduces the utilization of pesticides, but also seriously impacts the growth of sensitive crops in non-target areas and pollutes the ecological environment [2].

Deposition and drift models of UAV spraying droplets have been established through wind tunnel experiments and field experiments to predict the drift distance and deposition distribution under various meteorological conditions, as well as to evaluate according to weather station data whether a particular time is suitable for pesticide spraying to help select the appropriate operational parameters for UAV spraying [3,4,5,6]. However, these models have poor real-time performance. In other words, there is no corresponding improvement measure for the droplet drift problem resulting from crosswind outburst and local climate changes. Hence, it is imperative to spray accurately based on the real-time wind speed and direction of the spraying area in the entire pesticide spraying process. Affected by the downwash generated by the UAV, the airborne meteorological sensor system might present inaccurate data, despite still being capable of monitoring weather variations in the working area in real time. Moreover, the airborne meteorological sensor system, in general, is mounted on the manned aircraft with favorable loading capacity. The crop protection UAV, however, possesses a limited net payload since it is already carrying such facilities as spraying kits and pesticides. Clearly, equipment and methods that can provide real-time and accurate meteorological data for UAV spraying, without giving the UAV additional burden, are needed.

The wireless sensor network has been vigorously developed in the field of precision agriculture such as crop condition monitoring, precision fertilization and irrigation. With the cost decline in various agricultural sensors, crop growth condition monitoring will certainly be popularized in all stages of agricultural production. Environmental data collected in the working area can be sent to the UAV in real time by the wireless sensor network. Then, the UAV can reduce pesticide drift through adaptive route adjustments according to changes in meteorological conditions. Moreover, leaf wetness sensors, which detect the distribution of droplet deposition, can feedback the spraying effect to the UAV and help in ensuring accurate spraying [7].

Rapid development has been witnessed in research concerning the UAV-WSN system in recent years. In UAV-WSN systems, UAVs mainly assist WSN in performing the following four functional categories:

- Collecting sensor node data as a relay or mobile sink. UAVs help in performing time-sensitive tasks [8,9,10,11], monitoring emergencies [12], saving energy of WSN [13,14,15,16,17], extending the lifetime of WSN network [14,18], connecting separate nodes and networks [12,19], and enabling data aggregation [17,20]. Measures such as optimizing the flight routes [9,12,14,15,16,19] and locations [15,19,21] of UAVs, designing clustering mechanisms [17,18,22], media access control mechanisms [10,18,23,24], and sleeping schedules of WSN [14] and the like have been adopted to achieve these targets.
- Assisting in locating the WSN nodes [25,26,27].
- Performing wireless charging for the WSN nodes [28,29,30].
- Dispersing the WSN nodes in large areas [31].

On the other hand, the WSN enable UAVs to operate better in different fields. Di Gennaroa et al. [32] proposed the use of micro UAVs equipped with multispectral cameras along with WSN meteorological data to evaluate the correlation between grapes quality and measured values. Based on the data sensed by the wireless sensor network deployed in the crop field, an architecture [33] and a computer-based system approach [34], which adjust the plant protection UAV route to changes in wind intensity and direction, were evaluated. Experimental results showed they can provide a more accurate deposition of pesticides. Droplet deposition was monitored [33] by setting sensors on both sides of the working area in such a way that the UAV route can be adjusted at intervals according to the difference in the concentration of droplet deposition until droplets on both sides are evenly distributed. Trajectory optimization was used [34] to minimize the difference between the total amount of pesticide used and the total amount of pesticide deposited in the target area. Note that it is difficult to acquire the total amount of pesticide deposited in the target area, since calibrating the waiting time for the pesticide droplet depositing onto the sensor is difficult. Assuming that the waiting time is long enough to obtain the accurate amount of deposition, there is still the issue of delay that needs to be dealt with during the real-time trajectory adjustment. Moreover, it is also challenging to determine how to convert the deposition volume sampled from the sensor node into the amount of pesticide deposited in the entire target area. On top of these, in the above cases, environmental pollution drift models were applied for simulating the deposition of pesticide droplets during verification, when it should have been a droplet drift model that was taken into account for UAV pesticide spraying.

With the view to controlling pesticide drift, this study consults previous research findings to design a system that adjusts the UAV flight route in line with the wind direction and wind speed monitored by the ground wireless sensor network. Contributions made by this study are summarized as follows:

- Pesticide drift was controlled by optimizing the spraying route of the crop protection UAV using deep Q-network (DQN) and particle swarm optimization (PSO).
- A pesticide droplet drift model that includes wind speed and wind direction as parameters was developed and adopted to simulate and compute the drift distance of pesticide droplet.
- Communication latency between the UAV and WSN has been measured and proven that it can meet the real-time requirements of the system.
- A LSTM-based wind speed prediction model and an RNN-based wind direction prediction model were established to deal with the problem of failing to acquire the latest wind speed and direction data due to the delayed communication or temporary disconnection with the ground node.
- An embedded development system was built to run the wind speed and wind direction prediction models as well as the PSO trajectory optimization algorithm based on the Raspberry Pi 4B+. It has also been proven that this approach can satisfy the real-time requirements of the practical application.

The rest of this paper is organized as follows. In Section 2, based on the simplified UAV pesticide drift model, the DQN-based and PSO-based UAV trajectory adjustment schemes were proposed. Meanwhile, a short-term wind speed and direction prediction model was also presented. This is followed by the assessment that DQN-based and PSO-based UAV trajectory adjustment schemes can potentially be applied in pesticide drift control. In Section 3, the simulation of trajectory adjustment algorithms based on DQN and PSO were described and discussed, and the effectiveness of the wind speed and direction prediction model was analyzed as well. Hardware testing and implementation were presented and discussed in Section 4. A final section summarizes the conclusions and proposed directions for future research.

## 2. System Model

**Figure 1:**
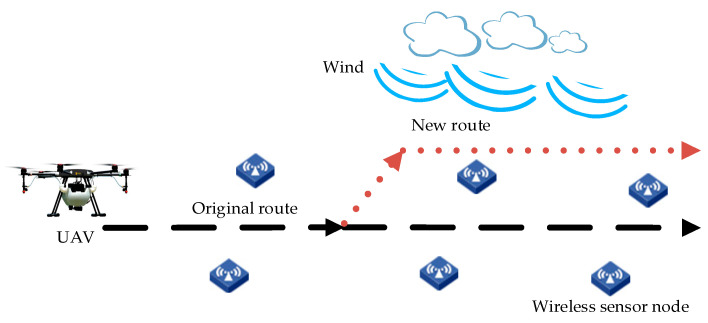
UAV spraying route changed based on ground sensor data.

Due to the drifting effect of natural wind, it is possible for pesticide droplets to be blown out of the target area, leading to not only poor droplet deposition within the target area, but also pollution to the surrounding environment. In response to this problem, a UAV trajectory adjustment scheme has been proposed, as shown in Figure 1. The plant protection UAV is equipped with a communication module (e.g., a ZigBee coordinator), which enables data collection from ground WSN arranged on the crop fields. While spraying, the UAV optimizes the spraying route according to the real-time wind speed and wind direction presented by ground sensor nodes. The black dashed line in the figure indicates the scheduled UAV spraying trajectory (normally, it is the central axis of the operation zone). In the case of sudden wind gusts, the UAV adjusts its flight trajectory by turning at a track angle to a certain side, deviating from the central axis (the red dotted line in the figure). When the displacement (the vertical distance between the UAV position and the original route) is adjusted to a proper value, the UAV returns to the start track angle and flies parallel to the central axis. By this means, the pesticide deposition is more balanced in the target area. During the trajectory adjustment process, pesticide spraying isn’t interrupted.

**Figure 2:**
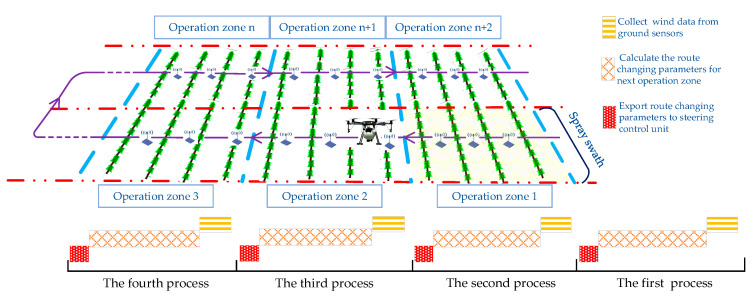
Operation environment and data process of the spraying UAV.

It is assumed that a crop field is divided into adjacent operation zones by taking the UAV spray swath as the width of each operation zone and the straight-line distance of the UAV flying in a route changing period as the length of the operation zone. In this study, the route changing period is set as 10 s, so as to match the wind change on real time basis. Figure 2 shows the steps to acquire route changing parameters by the UAV while spraying in an operation zone: (i) Collecting the wind speed and wind direction data from the ground sensors at 10 s intervals; (ii) Calculating the route changing parameters for the next operation zone based on the sensor data with the algorithms DQN or PSO; (iii) Exporting the route changing parameters to the steering control unit of the UAV. This process runs sequentially for each operation zone until the end of the spraying process.

When the spraying starts in an operation zone, the UAV sends a broadcast message to the ground sensor nodes, requesting the wind speed and wind direction. In the case of long operation zone, querying the sensor nodes located in the next operation zone is needed and can be performed by using multi-hop links wireless communication between UAV and ground WSN. As the operation zone is relatively short in this research (the distance that a plant protection UAV can fly in 10 s with a typical speed from 5 m/s to 10 m/s), the wind data from the sensor node in the current operation zone is also suitable for the next operation zone trajectory adjustment. The wind speed and wind direction are sampled every 1 s. At the other times, ground sensor nodes switch into a sleeping state and regularly wake up for messages. The sensor nodes that receive the UAV message transmit the latest wind data to the UAV in one hop. The UAV then takes the average of the acquired sensor data to obtain the wind information. By this means, communication delay can be decreased compared with the multi-hop transmission. To make sure at least one sensor node can respond to the UAV’s requests at any time, the distance between adjacent nodes in an operation zone or among neighboring zones is set as nearly 50 m for a network of the ZigBee protocol, while the node sleep time is set as 30 ms. On receiving the wind data, the DQN algorithm or PSO algorithm are applied to generate the route changing parameters for the next operation zone, which are illustrated in detail in Section 2.1 and Section 2.3. When the spraying of the next operation zone starts, the UAV updates its flight trajectory according to the route changing parameters, and in the meantime, another cycle of acquiring the adjustment parameters is executed.

There are two constraint conditions for successfully executing the route changing process. One important condition is that the computational cost (runtime) of acquiring the route changing parameters should be lower than the time required for spraying a single operation zone. The other condition is that the wind data should be received by the UAV in time. In the event of packet loss that prevents the timely wind information from being received by the UAV, two backup settings can be used: (i) In the case of wind data missing in a short time, the wind speed and direction is predicted based on the previously received sensor data, which will be discussed in Section 2.4. (ii) In the case of wind data missing in a longer time, the UAV keeps the spraying trajectory to the default route (it is mostly the central axis of the operation zone).

### 2.1. The DQN-Based UAV Trajectory Adjustment Scheme

Served by the data from field wireless sensors, the crop protection UAV accepts droplet drift and deposition data as feedback and adjusts its flight route in real time based on the wind speed and wind direction data of the farmland during the spraying process. In this approach, a closed control loop is formed, which requires a mechanism to collect the environmental status information (wind speed and direction). This can also be analogized to accumulating experience through learning and exploration, before gradually adjusting the strategy (i.e., spraying trajectory) through environmental feedback (i.e., droplet deposition).

Reinforcement learning [35] is a learning mechanism that is executed via the interaction between the agent and the dynamic environment. It evaluates in accordance with the feedback of the environment on the actions taken by the agent subject to guide the subsequent actions, so that good actions can be reinforced. This implies that a satisfactory action strategy can be acquired through trials to adapt to the environment. DQN [36] approximates value functions using the deep learning technique compared with the traditional reinforcement learning method. By doing so, it can not only alleviate the curse of dimensionality caused by the excessively large state and action space, but also address the unstable function approximation in RL using experience replay and a destination network.

#### 2.1.1. Working Environment

The UAV employs cattle ploughing reciprocation and is capable of spraying the whole farmland with an effective spraying width. The default spraying trajectory is set along the central axis of each operation zone, with wind speed and direction sensors arranged in each operation zone as needed. If feedback on droplet deposition is needed from the sensor during reinforcement learning, both sides of the central axis in the operation zone will be configured with two rows of wireless sensor nodes and leaf wetness sensors at equal distances. If the droplet deposition feedback is not needed, then the wind speed and direction sensors will be arranged as desired, rather than placed in symmetry.

#### 2.1.2. States

UAV is an intelligent learning agent that can perceive its environment in multidimensional states. Therefore, it is important to select the states which have direct association with trajectory optimization effect for observation. However, if too many states are selected, an excessively large state space will be formed, leading to calculation overload and reducing the convergence speed. If insufficient states are selected, some of the important environment information will be lost, hindering the UAV from learning the optimal strategy. The following state spaces were constructed on the basis of the acquirable sensor data:

(1) $$S=\left(U,X_{w},\theta_{w}\right)$$

where *U* represents the position of the UAV, $X_{w}$ the wind speed, and $\theta_{w}$ the wind direction. The wind speed and wind direction are delivered to the UAV via ground sensor nodes, whereas the position statuses are obtained by the GPS and built-in sensors of the UAV.

#### 2.1.3. Action

During the reinforcement learning, the agent selects an action from the set of optional actions according to a certain strategy to be executed upon observing the current states (wind speed, wind direction, and position) of the environment. The determination of the optional action space exerts a crucial influence on the agent’s learning process. In this study, the UAV action is to advance a step by selecting a certain steering angle at the current position and to fly in parallel with the central axis of the working area after adjusting to an appropriate position (as shown in Figure 1). In view of the spraying width and steering characteristics of the crop protection UAV, if the steering angle is too large, the UAV will move too far away from the central axis in a step, resulting in the fact that the route adjustment is not precise, and it may even move the UAV outside the operating area. Therefore, a small angle of –15° to +15° is applied to fine-tune the deviation of the UAV. Since at each step the DQN algorithm calculates the Q values for all actions in the action space, overlarge action space will cause too many output units, increasing the calculation time and weakening the recognition ability of the deep neural network. Therefore, discrete steering angles were selected rather than continuous angles [37] to form the action space. The difference between the angles is 5 degrees, which is sufficient for the application of plant protection UAVs. The action space was designed based on 7 steering angles, as shown below:

(2) A={−15°,−10°,−5°,0°,5°,10°,15°}

By doing so, it can not only reduce the complexity of the neural network approximator of the action value function, but also improve the timeliness of planning.

#### 2.1.4. Reward

The action feedback of UAV is acquired by means of environmental reward functions in the process of deep reinforcement learning. The reward function has a direction impact on the performance and the convergence speed of the reinforcement learning algorithm since it implicitly defines the learning target. When the UAV sprays down at a farmland distributed with leaf wetness sensors, it will receive droplet deposition data on both sides of the central axis in real time while spraying. When the difference of the droplet deposition concentration on both sides is greater than a certain threshold *T*, the pesticide droplets in that area will be regarded as having drifted towards a certain side of the work area. Since reinforcement learning is targeted at droplet deposition as uniformly as possible in the targeted work area, the reward can be given in accordance with the difference ∆c in droplet deposition concentrations read by the leaf wetness sensors on both sides of the central axis. The specific definition of reward in this paper is shown in Equation (3):

(3) r=exp[−1.3×(Δc−T)]−1

where T is the predefined threshold. If ∆c < *T*, the agent will obtain a positive reward, which will increase with the decrease of ∆c. If ∆c < *T*, the agent will obtain a negative reward, and if ∆c = *T*, zero reward will be given. This design ensures the continuity of the reward function and accelerates the convergence speed of the algorithm. It is worth noting that, since the deposition amount on the sensor increases with observation time, the difference ∆c in droplet deposition concentration on both sides of the central axis will also be proportional to the monitoring time after the droplets spraying, both of which affects the determination of the threshold *T*. Hence, the threshold should be practically calibrated through experiments.

For environments not distributed with leaf wetness sensors, the distance d between the center of droplet deposition, which can be computed by the droplet drift model, and the central axis of the work area can be used to indicate the immediate reward, which can be defined as Equation (4):

(4) $$r=\left\{ \begin{array}{l} -a,\;\;\;\;\;\;\;\;\;\;\;\;\;\;\;\;\;\;\;\;\;\;\;\;\;\;\;\;\mathrm{if}\;d>I\\ \exp\left(1.3d\right),\;\;\;\;\;\;\;\;\;\;\;\;\;\;\;\mathrm{if}\;d\leq I \end{array}\right.$$

where *d* is the vertical distance between the center of droplet deposition and the central axis of the working area, *I* (*I* > 0) is the set threshold, and *a* (*a* > 0) is a constant. Algorithm 1 shown below describes the DQN algorithm used for the UAV trajectory adjustment.
**Algorithm 1** DQN-based UAV Trajectory Adjustment1:**Initialize**: Initialize an experience replay memory with capacity *D*2:      Initialize the online network *Q* with random weight ${\theta \;=\;\theta }_{0}$
3:      Initialize the target network *Q*′ with the weight ${\theta^{\prime }\;=\;\theta }_{0}$
4:**for** episode l = 1 to *M*
**do**5:  Initialize the activity state of UAV6:  **for** t = 1 to *T*
**do**7: 8:  According to current state $s_{t}$, with  probability ε  randomly select an action $a_{t}$, otherwise select $a_{t}{\;=\;\mathrm{argmax}}_{a}\;\left(s_{t\;},\;a_{t\;},\;\theta \right)$
9:  Receive reward $r_{t}$ by formula (4) and observe new state $s_{t+1}$
10:  Store transition ($s_{t\;}{,\;a}_{t\;},\;r_{t\;}{,\;s}_{t+1}$) in the replay memory *D*11:  Sample a random minibatch of transitions ($s_{i}\;{,\;a}_{i}\;{,\;r}_{i}\;{,\;s}_{i+1}$) from *D*12: 13: 14:  Set $y_{i}=\left\{ \begin{array}{l} r_{i},\;\;\;\;\;\;\;\;\;\;\;\;\;\;\;\;\;\;\;\;\;\;\;\;\;\;\;\;\;\;\;\;\;\;\;\;\;\;\;\;\;\;\;\;\;\;\;\;\mathrm{if}\;\;\mathrm{terminal}\\ r_{i}+\gamma \max_{a^{\prime }}\;Q\prime \left(s_{i+1},a^{\prime };\;\theta \prime \right),\;\;\;\;\mathrm{otherwise} \end{array}\right.$
15: 16:   Calculate the loss ${\left(y_{i}-Q\left(s_{i},a_{i};\theta \right)\right)}^{2}$
17:  Perform a gradient descent step on the loss function with respect to  θ
18:   Train and update Q network weights θ19:   Every C step set ${\theta^{\prime }\;\leftarrow \;\theta }_{0}$
20:  
$s_{t\;}\leftarrow {\;s}_{t+1}$
21: 
**end for**
22:**end for**

#### 2.1.5. Neural Network Approximation of DQN Value Function

**Figure 3:**
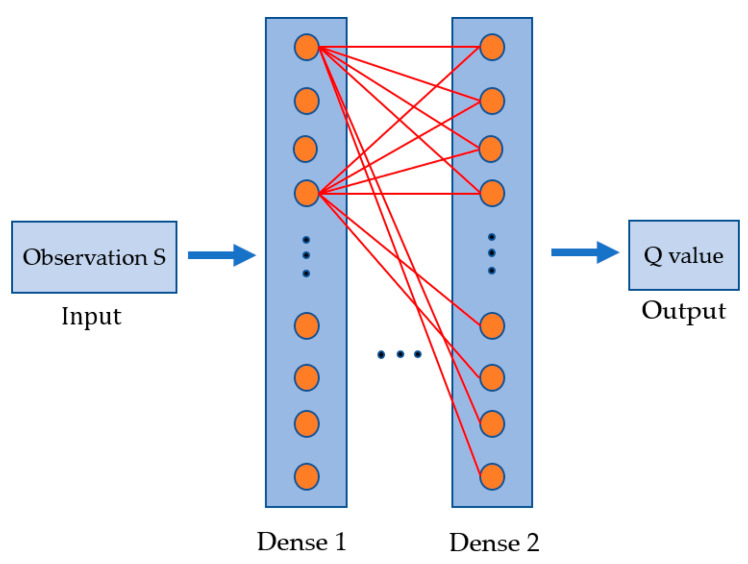
Neural network based value function approximator of Reinforcement learning.

DQN is a reinforcement learning method that approximates the value function Q using a neural network. The neural network structure constructed in this paper, as shown in Figure 3, is composed of two fully connected layers instead of the convolutional layer in the common deep Q network. Moreover, a rectified linear unit (ReLU) is used as the activation function, and the number of neurons in each layer is 32. Its input is the state $S=\left(U,X_{w},\theta_{w}\right)$, and its output is the corresponding Q value.

### 2.2. The Simplified UAV Pesticide Drift Model

The droplet drift model, which focuses on the drift distance of pesticide droplets under the influence of various factors, has important guiding significance for adjusting the pesticide spraying trajectory in this study. Rotor wind field, UAV flight height and speed, wind speed and direction as well as droplet size and nozzle pressure are critical factors affecting droplet drift. Because this study is focused on the method of adjusting the spraying route based on real-time wind speed and direction, a simplified droplet drift model that depends primarily on wind speed and direction was proposed, while the UAV model, flight height and speed, as well as the droplet size and nozzle pressure were set as fixed options.

The regression equation for the drift distance of the droplet deposition center obtained via wind tunnel experiments [5] can be expressed as:

(5) $$Y=0.167X_{w}+0.085X_{p}+0.308X_{h}-0.667,\;\;\;\left(R^{2}=0.774\right)$$

where $X_{w}$ (m/s) is the wind speed, $X_{p}$ (Mpa) is the nozzle pressure, $X_{h}$ (m) is the spraying height, *Y* (m) is the drift distance of the droplet deposition center, and $R^{2}$ is the coefficient of determination. 

**Figure 4:**
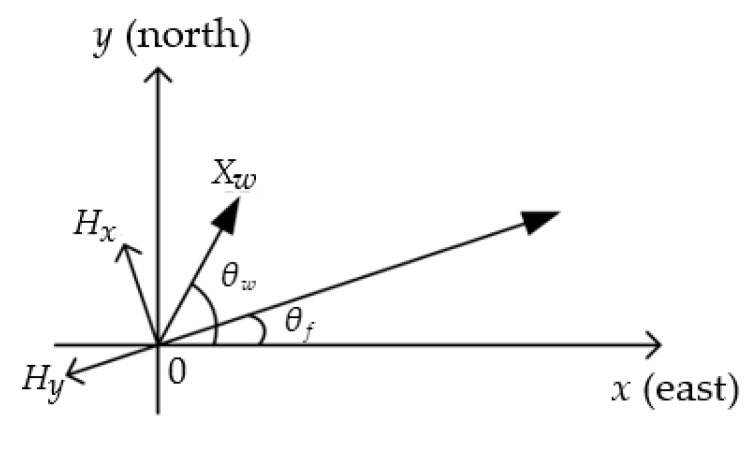
Schematic diagram of flight direction, wind direction, and deposition components.

It can be seen from Figure 4 that the coordinate system is set with the UAV position as the origin, the positive axis of the x axis as the direction of due east, and the positive axis of the y axis as the direction of due north. In this study, the height was excluded. With regards to various position and distance parameters, only their projections on the  xy coordinate plane were taken into account. Assuming that $\theta_{w}$ is the angle formed between the wind direction and the positive axis of x, and $\theta_{f}$ is the angle between the flight direction and the positive axis of x, performing an orthogonal decomposition on $X_{w}$ will obtain the drift distance component $H_{x}$ of the droplet deposition center in the vertical to the UAV flight direction, and the drift distance component $H_{y}$ of the droplet deposition center along the UAV flight direction.

(6) $$H_{x}=0.167X_{w}\sin\left(\theta_{w}-\theta_{f}\right)+0.085X_{p}+0.308X_{h}-0.667$$

(7) $$H_{y}=0.167X_{w}\cos\left(\theta_{w}-\theta_{f}\right)+0.085X_{p}+0.308X_{h}-0.667$$

The above conclusion describes the influence of wind speed on the drift of the droplet deposition center when the UAV is hovering. Since the actual flying operation of the UAV should be considered in this study, the influence of the flight speed of UAV on the droplet drift should be also taken into account. Notice that pesticide droplets drift toward the opposite of UAV flight direction. For a M234-AT model quadrotor fitted with a pressure nozzle made by Lechler GmbH (Metzingen, Germany), the relationship between the drift and the distance behind the UAV, given that the nozzle position is 0.5 m, the distance between nozzles is 0.25 m, the flight height is 1.5 m, and the flight speed 6 m/s, can be expressed as [38]:

(8) $$y=41.34-22.22\log\left(x\right),\;\;\;\left(R^{2}=0.991\right)$$

where y represents the percentage of the drift behind the UAV to the total drift, in percentage; x represents the distance to the rear of the fuselage, in meters. Furthermore, Equation (8) can be rewritten as:

(9) $$x=\exp\left(\frac{41.34-y}{22.22}\right)$$

Using the above equation, x = 0.677 is computed upon substituting y = 50 into the expression. That is to say, the amount of droplet deposition within the range of 0.677 m behind the fuselage under windless conditions can reach up to 50% of the total deposition amount. Taking Equation (7) into account, the drift distance component of the droplet deposition center directly behind a flying M234-AT quadrotor under windy conditions can be expressed as:

(10) $$H_{y}=0.167X_{w}\cos\left(\theta_{w}-\theta_{f}\right)+0.085X_{p}+0.308X_{h}-0.667-0.677$$

Substituting nozzle pressure $X_{p}$ = 1 MPa and spraying height $X_{h}$ = 1.5 m into Equations (6) and (10), $H_{x}$ and $H_{y}$ can be rewritten as:

(11) $$H_{x}=0.167X_{w}\sin\left(\theta_{w}-\theta_{f}\right)-0.12$$

(12) $$H_{y}=0.167X_{w}\cos\left(\theta_{w}-\theta_{f}\right)-0.797$$

from the above expression, the drift distance components of the droplet deposition center in the positive x axis direction and the positive y axis direction are:

(13) $$x=H_{x}\sin\theta_{f}+H_{y}\cos\theta_{f}$$

(14) $$y=H_{x}\cos\theta_{f}+H_{y}\sin\theta_{f}$$

Then, substituting Equations (11) and (12) into Equations (13) and (14), the simplified pesticide droplet drift model can be obtained:

(15) $$D\left(X_{w},\theta_{w},\theta_{f}\right):\left(\begin{array}{l} x=0.167X_{w}\cos\left(\theta_{w}-2\theta_{f}\right)-0.12\sin\theta_{f}-0.797\cos\theta_{f}\\ y=0.167X_{w}\sin\theta_{w}-0.12\cos\theta_{f}-0.797\sin\theta_{f} \end{array}\right)$$

where D$\left(X_{w\;}{,\theta }_{w}\;{,\theta }_{f}\right)\;$ represents the relative distance of the droplet deposition center to the UAV under wind speed ${\;X}_{w\;}$, wind direction ${\;\theta }_{w}$, and UAV flight direction ${\;\theta }_{f}\;$.

### 2.3. UAV Trajectory Adjustment Based on the PSO Algorithm

Particle swarm optimization is a kind of self-adaptive random algorithm based on group hunting strategy featuring fast convergence rate, as well as easiness in programming and implementation since the optimized function is not required to be differentiable, derivable, and continuous. It is hence perfect for solving a high-dimensional UAV trajectory optimization problem that demands high real-time performance but tolerates lower-precision solutions.

**Figure 5:**
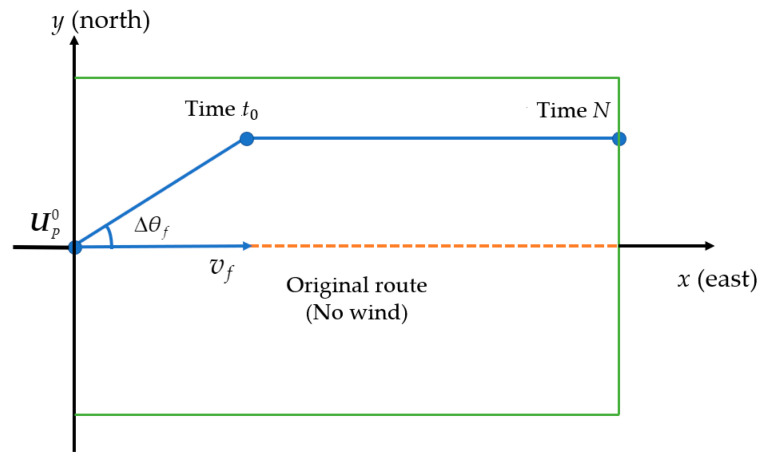
The principle of PSO-based trajectory adjustment.

As can be seen from Figure 5, a coordinate system is established with the starting point of the central axis in the current work area set as the origin of the coordinates. The due East and due North directions are respectively represented with the x axis and y axis to match with the pesticide drift model. Without a loss of generality, the central axis of the work area is assumed to coincide with the due east direction in Figure 5. The initial position of UAV in the current trajectory adjustment cycle is set as $U_{P}^{0}$. $U_{P}^{0}$ = (0, 0) is assumed in Figure 5. If the trajectory is not adjusted, the UAV flies and applies pesticide at the speed of ${\;v}_{f\;}\;$ along the central axis of the work area or in a direction parallel with the central axis. Given the central axis is assumed to coincide with the due east direction, thus the angle $\theta_{f}$ formed by the flight direction and the due east direction is 0° in Figure 5. The UAV spraying position at every t(t∈N) can be expressed by:

(16) $$U_{P}^{t}=U_{P}^{0}+G(v_{f},\theta_{f},t)$$

where ***G***${(v}_{f\;}{,\;\theta }_{f}\;,t)$ represents the distance of the UAV moving in a XY coordinate plane during time period of t . Note that the distances and positions discussed in this section are those of XY coordinate plane, excluding the height. Moreover, the distance between the droplet deposition center and the UAV is ***D***($X_{w\;}^{t}{,\theta }_{w}^{t}\;{,\theta }_{f}$) upon computing with the droplet drift model mentioned in Section 2.2, where $X_{w}$ is wind speed and $\theta_{w}$ is wind direction. Then, under windy conditions, the position $P^{t}$ of the droplet deposition center of the sprayed pesticide droplet at time t can be calculated as

(17) $$P^{t}=U_{P}^{0}+G(v_{f},\theta_{f},t)+D(X_{w}^{t},\theta_{w}^{t},\theta_{f})$$

The UAV sprays pesticide along the central axis of the work area is the optimal strategy under windless conditions. In this scenario, the position of the deposition center point of the sprayed pesticide droplet at time t is

(18) $$P_{0}^{t}=G(v_{f},\theta_{f},t)+D(0,0,\theta_{f})$$

This position is the optimal position for droplet deposition at time t. Under windy conditions the central points of the droplet deposition would drift out of these optimal positions, the flying angle and speed of the UAV should be adjusted in a manner shown in Figure 5. Assuming that the UAV turns to $∆\theta_{f}$ from the original flight direction with increased flight speed $∆v_{f\;}$, and the rotational direction maintained for time period of $t_{0\;}$, upon resuming a flight direction that is parallel with the central axis and the original flight speed $v_{f\;}$ at the end of the turn. When $∆\theta_{f}$ is positive, it indicates that the UAV is deflected northeast, and when it is negative, it indicates that the UAV is deflected southeast.

The target of PSO-base trajectory optimization is to minimize the distance sum between the droplet deposition centers of spraying along the new route and the droplet deposition centers of spraying along the central axis under windless conditions at the sampling moments. In this study, the sampling interval is 1 s, and the range of N seconds is a cycle for route adjustment. The trajectory optimization function is expressed as

(19) $$\begin{array}{l} \;\;\;\;\;\;f(\Delta v_{f},\Delta \theta_{f},t_{0})\\ =\underset{\Delta v_{f},\Delta \theta_{f},t_{0}}{argmin}\sum_{t=1}^{N}\left\|P^{t}-P_{0}^{t}\right\|\\ =\underset{\Delta v_{f},\Delta \theta_{f},t_{0}}{argmin}\left\{\begin{array}{l} \sum_{t=1}^{t_{0}}\|U_{P}^{0}+G(v_{f}+\Delta v_{f},\theta_{f}+\Delta \theta_{f},t)+D(X_{w}^{t},\theta_{w}^{t},\theta_{f}+\Delta \theta_{f})-G(v_{f},\theta_{f},t)-D(0,0,\theta_{f})\|+\\ \sum_{t=t_{0}}^{N}\|U_{P}^{0}+G(v_{f}+\Delta v_{f},\theta_{f}+\Delta \theta_{f},t_{0})+G(v_{f},\theta_{f},t-t_{0})+D(X_{w}^{t},\theta_{w}^{t},\theta_{f})\;-G(v_{f},\theta_{f},t)-D(0,0,\theta_{f})\| \end{array}\right\} \end{array}$$

The trajectory fine-tuning model can be regarded as a function expression whose independent variables are $∆v_{f\;},\;∆\theta_{f}\;,{\;t}_{0\;}\;$ and search space dimension D = 3.

Algorithm 2 describes the optimization process. First, by initializing the particle swarm, each particle has a random initial position and initial speed. Then, the maximum number of iterations of the algorithm is set. A possible solution should be detected when the maximum number of iterations or the given conditions is met. Fitness values of all particles are obtained through optimizing the function *f*$\;(∆v_{f\;},\;∆\theta_{f}\;,{\;t}_{0\;}$). At any instance during the iteration, when the fitness of a particle is the optimal value obtained among the completed iterations, the particle position is saved as the optimal in history. When the fitness of a particle is the optimal value of all particles, the particle position is saved as the optimal in the swarm. An optimal solution or a sub optimal will be obtained by the end of the algorithm iteration.
**Algorithm 2** PSO-based UAV Trajectory Adjustment1: Initialize particles (random velocities and positions for particles)2: **for** iteration = 1 to  Max_iterations
**do**3:    **if** expected conditions are met **then**4:      **break**5:    **end if**6:    **for** each particle *i*
**do**7:       Calculate the fitness value according to the function (19)8:       **if** the fitness value is the best of the particle **then**9:         Store the position of the corresponding particle in the particle10:      **end if**11:      **if** the fitness value is the best of the swarm **then**12:        Store the position of the corresponding particle in the swarm13:      **end if**14:    **end for**15:  Update the particles’ velocities and positions16: **end for**17: Output the best particle

### 2.4. Establishment of a Short-Term Wind Speed and Direction Prediction Model

The DQN and PSO algorithms proposed in this paper perform trajectory optimization in accordance with wind speed and direction data sent by the ground wireless sensor network to the UAV. Nevertheless, isolated nodes and networks might occur due to the large farmland area, causing the UAV to fail to receive the wind speed and direction data. Moreover, large communication latency might be caused by re-transmission because of packet loss, affecting the real-time operation of the trajectory adjustment mechanism. At this point, a method that can be used for quickly predicting the wind speed and direction is required. For this reason, a short-term wind speed and direction prediction model is established to predict the wind speed and direction in the area to be sprayed when the communication latency becomes large.

Based on the collected wind direction and wind speed data, LSTM wind speed prediction and RNN wind direction prediction were selected in this study after comparing the wind speed and direction prediction effects of a fully connected neural network, one-dimensional convolutional neural network, recurrent neural network (RNN), and long and short-term memory network (LSTM). 

The wind speed and wind direction prediction model is designed into a three-input and single-output network structure, and its inputs are two-dimensional tensor composed of wind speed and wind direction data, as well as average humidity data and average temperature data.

The wind speed prediction model consists of wind speed and direction feature extraction layer, humidity feature extraction layer, temperature feature extraction layer, feature stitching layer and wind speed vector prediction layer. The feature extraction layer is comprised of an LSTM layer and dropout layer. Specifically, the LSTM layer involving several neurons is used for extracting shallow features of sequence data. Dropout discards neurons, with the probability of p to break the contingency of feature combination, are used to transform shallow features of the sequence data. The feature stitching layer combines and compresses wind speed, wind direction, humidity and temperature features extracted by the feature extraction layer to obtain more abstract features. Finally, the wind speed sequence value over a short term in the future is computed via the wind speed vector prediction layer according to the features output by the feature stitching layer.

The wind direction prediction model consists of the wind speed and direction feature extraction layer, humidity feature extraction layer, temperature feature extraction layer, feature stitching layer, and wind direction vector prediction layer. The feature extraction layer is comprised of the RNN layer and dropout layer. The number of neurons in the RNN layer in the wind direction prediction model is twice the number of neurons in the LSTM layer in the wind speed prediction model. Similarly, compared with the wind speed prediction model, the wind direction prediction model also increases the number of neurons in the feature stitching layer without performing feature compression. The wind direction sequence value over a short term in the future is the output of the wind direction prediction model.

## 3. Results

In this section, the performances of two proposed trajectory adjustment algorithms are evaluated in simulation using the collected wind speed and direction data and the predicted wind speed and direction data, respectively.

### 3.1. Simulation of Trajectory Adjustment Algorithm of Crop Protection UAV for Pesticide Drift Control

**Figure 6:**
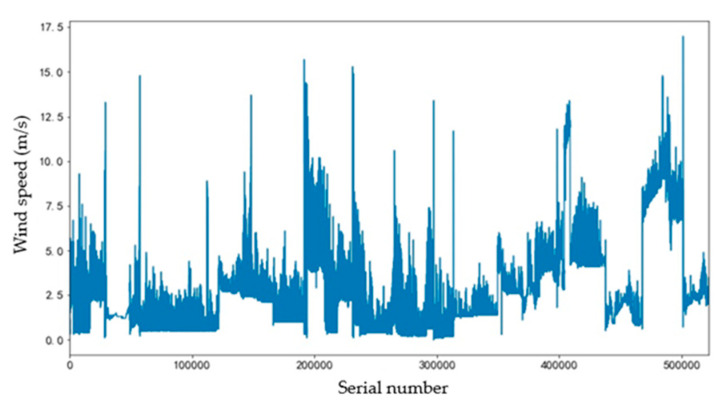
Wind speed data.

A STM32F103ZET6 single-chip microcomputer, a RS-FXJT05-V05 anemometer and an SD card were adopted for environmental data monitoring. The meteorological data of certain areas in the university from 20 June 2019 to 20 September 2019 were collected at the data collection cycle of 1 s. Parts of the collected wind speed data are shown in Figure 6. The accuracy of the anemometer is eight orientations.

10 s are set as the cycle period of trajectory adjustment. In the meantime, each 10 consecutive wind speeds and direction values are included in a set for one cycle period of trajectory adjustment. Table 1 is a set of data selected from the collected data with large changes, which are applied as wind speed and direction data for the following trajectory adjustment simulations.

#### 3.1.1. Simulation of DQN-Based UAV Trajectory Adjustment Algorithm

Concerning the reward design of the DQN algorithm, the concentration difference of the leaf wetness sensors on both sides of the central axis as mentioned in Section 2.1.4 can be adopted to express the reward function. Nevertheless, the relationship among the time of droplets depositing to the sensor node, the difference ∆c in the Equation (3) and the threshold T is not calibrated. Hence, the droplet drift model is adopted to replace the leaf wetness sensors to obtain the feedback of trajectory adjustment in verifying the effectiveness of the algorithm, namely, the reward of deep reinforcement learning is computed with the Equation (4). The initial position of UAV in the simulation is set as (0, 0.2), and the initial flight direction is due east. The UAV reaches the next state at a step size of 6 m. The threshold and the constant in Equation (4) are set as I = 1 m and a = 5, respectively. DQN training parameters are presented in Table 2. In this study, the DQN network architecture was developed with Python and implemented on TensorFlow. The simulations were carried out under Windows 10 system and the model of the processor is AMD Ryzen 5 2500U (2 GHz).

**Figure 7:**
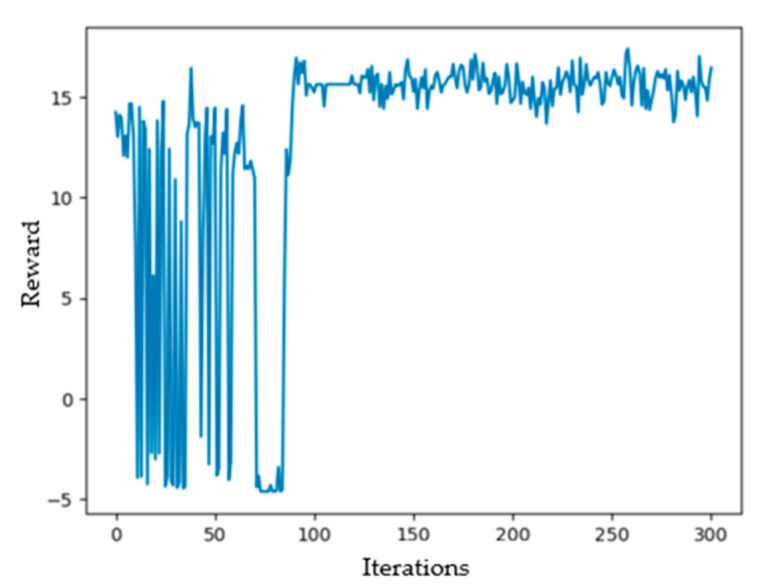
DQN cumulative rewards.

After 300 times of training, the curve of cumulative reward value with the number of training is shown in Figure 7. The convergence speed of the DQN algorithm is the key factor influencing whether the algorithm meets the real-time requirement of the UAV route changing scheme. It can be seen from the figure that the cumulative reward value tends to be stable when the number of iterations reaches about 85, indicating the fast convergence of the proposed DQN algorithm. During training, the more positive reward values are obtained, the more correct the judgment made by the UAV. As shown in Figure 7, the cumulative reward varies between −5 to 15 during the early stage of iteration, which illustrates that the agent (the UAV) is exploring and learning the action strategies, and inappropriate action will cause the cumulative reward value to decrease or even be negative. After multiple explorations, the agent can select the action with high reward values according to the status of the environment as it gradually understands the environment. As can be seen from Figure 7, when the iteration number reaches 85, the reward values obtained by the UAV no longer appear negative, indicating that the UAV can make correct judgment since then. In this study, the higher the cumulative reward value obtained indicates the fact that the closer the distance between the droplet deposition center and the target area during trajectory adjustment, the closer the trajectory approaching the optimal.

**Figure 8:**
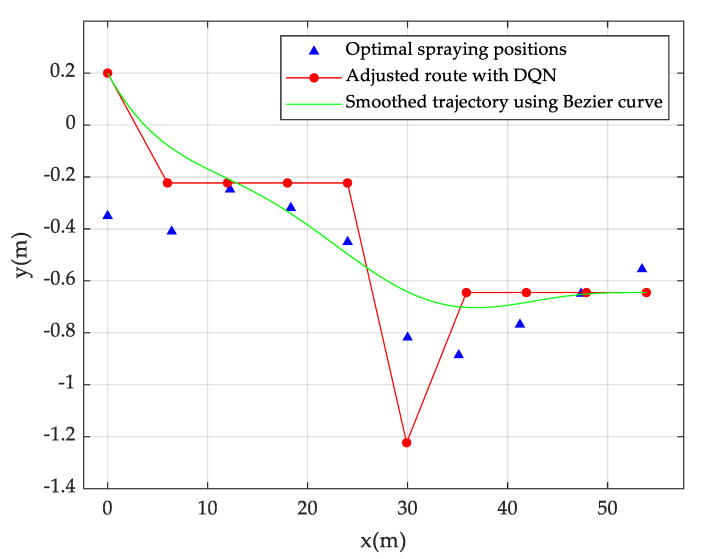
DQN-based UAV pesticide spraying trajectory adjustment.

After the algorithm convergence, the adjusted UAV spraying route is shown in Figure 8, of which, y = 0 in the figure is the central axis of the work area. The starting point is not necessarily located on the central axis, since the current starting point is the end point of the last trajectory adjustment. As can be seen from the figure, the blue triangles are the optimal spraying positions of UAV at each second, which are calculated with the pesticide drift model under the wind speed and direction data in Table 1. The dotted line in red is the spraying trajectory adjusted by the DQN algorithm. The trajectory shows that the UAV should make four steering adjustments in 10 time steps. Such an adjustment is too frequent to implement in the actual environment. Therefore, the Bezier curve [39] is adopted in this study to smooth the trajectory connecting the start point and the end point. The n-order Bezier curve is defined as Equation (20):

(20) $$Y(t)=\sum_{k=0}^{n}\left(\frac{n!}{k!(n-k)!}\right)Y_{k}t^{k}{\left(1-t\right)}^{n-k},\;\;\;t\in \left[0,1\right]$$

where *Y*(*t*) represents the Bezier curve; $Y_{k}$ represents the kth control point; and t represents the normalized time variable. In this paper, 10-order Bezier curve, i.e., n = 10 is adopted. The smoothed UAV route is shown in the green line in Figure 8.

#### 3.1.2. Simulation of PSO-Based UAV Trajectory Adjustment Algorithm

Wind speed and direction data in the table are also selected for algorithm verification in the simulation of the PSO-based UAV trajectory adjustment algorithm. The trajectory adjustment cycle N is set as 10 s. Fitness values of all particles are computed according to the trajectory optimization function *f* ($∆v_{f\;},\;∆\theta_{f}\;,{\;t}_{0}$) in Section 2.3. Parameter settings of the particle swarm algorithm are shown in Table 3. The value $∆v_{f\;}$ is assigned randomly –1 m/s ~ 1 m/s; $∆\theta_{f}$ is –π/6~π/6; and $t_{0}$ is 1 ~ 5 s in the initial particle position. $∆v_{f\;}$ is assigned randomly –0.2 m/s ~ 0.2 m/s, $∆\theta_{f}$ is –π/18~π/18, and ${\;t}_{0\;}$ is –1 s ~ 1 s at the initial particle speed. The inertia factor w is adaptively adjusted according to the current iteration number iter and the maximum number of iterations ${\mathrm{iter}}_{\max}$, as shown in Equation (21):

(21) $$w=\frac{0.5\times \left(iter_{\max}-iter\right)}{iter_{\max}}+0.4$$

$t_{0\;}$ of all particles should be checked after the particles have been updated in each iteration. If $t_{0}\;<\;0$, then it is set as zero. If $t_{0\;}\;>\;N$, then it is set as N, so as to prevent ineffective particles. In our simulation experiments, the PSO-based route changing algorithm was developed with Python and NumPy. We executed the simulation of PSO scheme on the Ubuntu 16.04 operation system with 2 GB NVIDIA GeForce GT 750 M graphics and Intel CORE i7-4500U (8 G).

**Figure 9:**
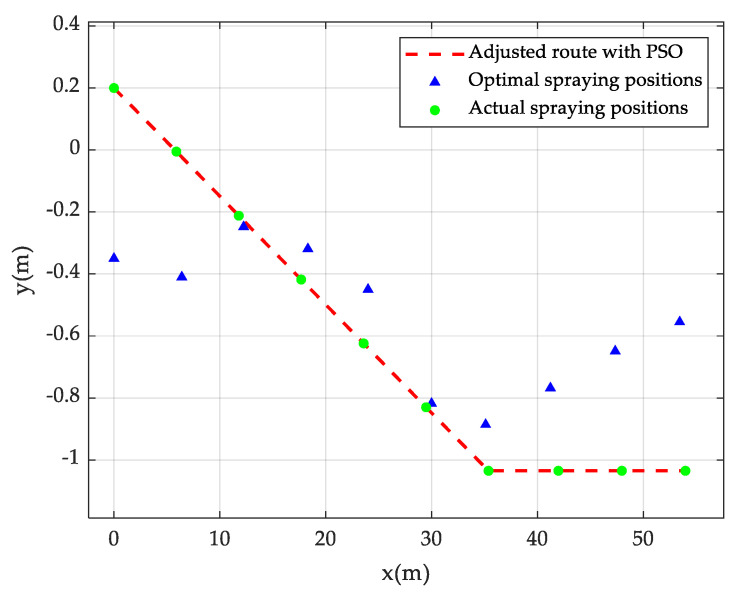
PSO-based UAV pesticide spraying trajectory adjustment.

Assume that the initial position of UAV is (0, 0.2); the flight speed is 6 m/s, and the flight direction is due east. This experiment is converged upon 9 rounds of iterations. Fine-tuned trajectory parameters obtained are $∆v_{f\;}\;=\;0.0\;m/s$, $∆\theta_{f}\;=\;-\pi /90$, and $t_{0}\;=\;7\;s$. Values of $∆v_{f\;},\;∆\theta_{f}\;,{\;t}_{0\;}$ and *f*$\;(∆v_{f\;},\;∆\theta_{f}\;,{\;t}_{0\;}$) in the iterative process are shown in Table 4, and the trajectory of pesticide spraying upon iterative convergence is shown in Figure 9. Similarly to Figure 8, the blue triangles represent the optimal spraying positions at each sampling time point under current wind speed and direction without considering the limitations in the UAV steering angle and steering times. The dashed line in red presents the optimal UAV spraying position upon the PSO-based pesticide spraying trajectory adjustment, and the green dots indicate the spraying position of each sampling time point on the trajectory optimized by the PSO algorithm.

#### 3.1.3. Comparison of DQN-Based and PSO-Based Trajectory Adjustment Effects

**Figure 10:**
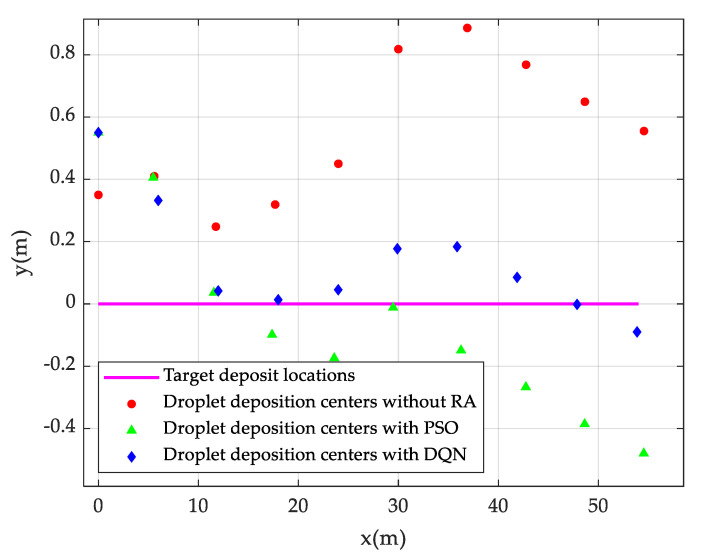
Positions of droplet deposition centers before and after route adjustments.

Droplet deposition centers obtained by DQN-based and PSO-based trajectory optimization methods are demonstrated in Figure 10. The straight line in purple represents the central axis of the work area, or the target position of the droplet deposition center. The red dot indicates the central positions of droplet deposition without route adjustment, i.e., the droplet deposition centers when spraying along the central axis. The blue rhombus presents the central positions of droplet deposition upon DQN-based route adjustment, and the green triangle represents the central positions of droplet deposition upon PSO-based route adjustment. It is observed that the deposition centers of pesticide droplets are approaching to the central axis of the work area upon adjusting the trajectory using two optimizing approaches, indicating that both approaches can effectively control pesticide drift under windy conditions. Based on the statistics from the figure, the sum of the vertical distances between the droplet deposition center and the target position (the central axis) upon route adjusting using the DQN algorithm and the PSO algorithm are 1.52 m and 2.558 m, respectively, in a route adjustment cycle. Whereas, when the UAV is spraying along the central axis, the sum of the distances between the position of the droplet deposition center and the target position is 5.453 m. Compared with no route adjustment, the DQN-based route optimizes the drift of the droplet deposition center points by 72.1%, and the PSO-based route optimizes by 53.1%.

### 3.2. Experiment on the Effectiveness of Trajectory Adjustment Based on the Predicted Wind Speed and Direction

This section examines whether trajectory adjustment using the predicted value of wind direction and speed can meet the requirement of accuracy. Among them, the predicted wind speed value is obtained by the LSTM-based model, and the predicted wind direction value is obtained by the RNN-based model. The wind speed and direction prediction model was developed with Python and implemented on TensorFlow. A graphics processing unit (GPU) was applied for neural network training and inference. We executed the simulation on the Ubuntu 16.04 operation system with 2 GB NVIDIA GeForce GT 750 M graphics and Intel CORE i7-4500U (8 G).

**Figure 11:**
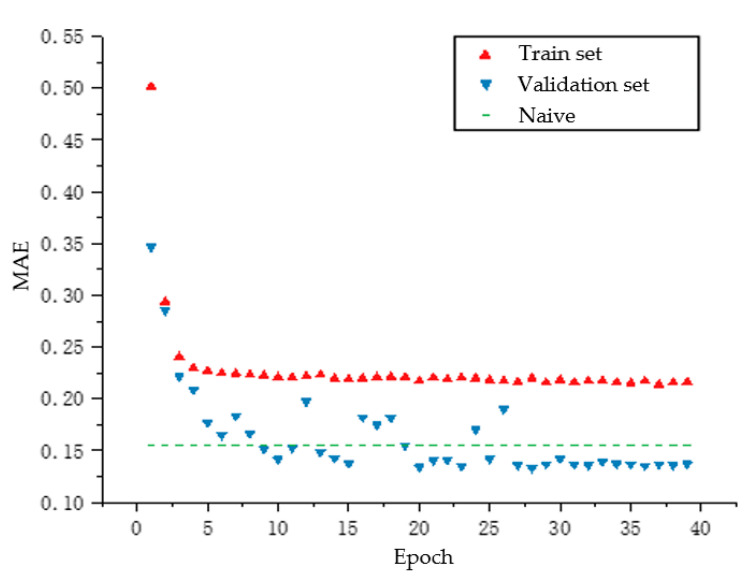
MAE indicator of the LSTM-based wind speed prediction model.

**Figure 12:**
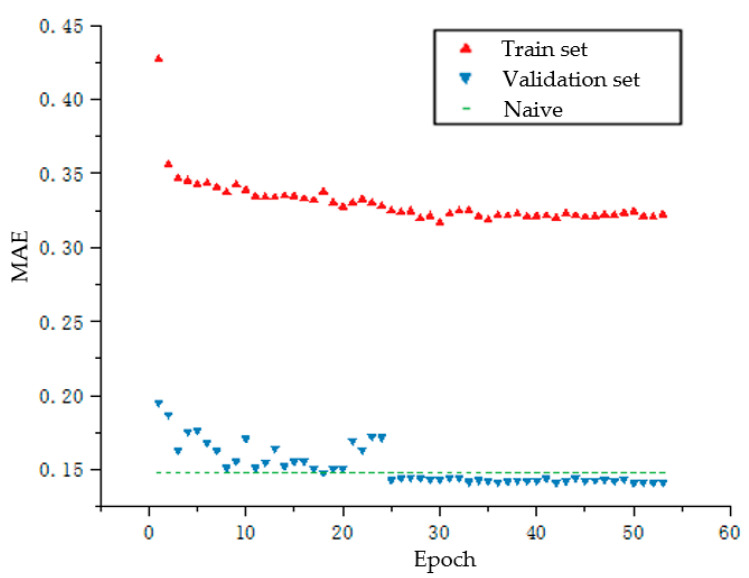
MAE indicator of the RNN-based wind speed prediction model.

Figure 11 shows the mean absolute error (MAE) indicator of the wind speed prediction model. Further, the MAE indicator of the wind direction prediction model is presented in Figure 12.

Next, the results of DQN-based and PSO-based trajectory optimization are compared with the following four types of wind speed and direction data:

- Route adjustment (RA) is not performed under the real wind speed and direction values, i.e., pesticide spraying along the central axis of the work area.
- Route adjustment is conducted by replacing the wind speed and direction values in the next 10 s with the current wind speed and direction value.
- Route adjustment is conducted with the predicted wind speed and direction values for the next 10 s.
- Route adjustment is performed with the predicted wind speed and direction values when the mean variation of wind speeds in the past 10 s is greater than the threshold. Otherwise, the current wind speed and direction value will take place of the wind speed and direction values in the next 10 s for trajectory adjustment.

The mean variation of wind speeds refers to the average sum of the difference of the wind speed minus the average wind speed per second in the past 10 s. According to changes in collected wind speed and direction data, the threshold W is set as 0.35 m/s, 0.5 m/s, and 1 m/s. The distance sum of the droplet deposition center of pesticide spraying along the new route in the trajectory adjustment cycle and the droplet deposition center under windless conditions is selected as the measuring indicator in the experiment, denoting as spraying accuracy f.

(22) $$f=\sum_{t=1}^{10}\left\|P^{t}-P_{0}^{t}\right\|$$

where $P_{0}^{t}$ is the droplet deposition center of pesticide spraying along the central axis at the time of t under windless conditions, and $P^{t}$ is the droplet deposition center of pesticide spraying along the corrected trajectory at the time of t under windy conditions. When evaluating the f value, 1000 sets of data randomly selected from the collected wind speed and direction data were set as the test data set. Moreover, ten wind speed and direction values are included in a trajectory adjustment cycle.

**Figure 13:**
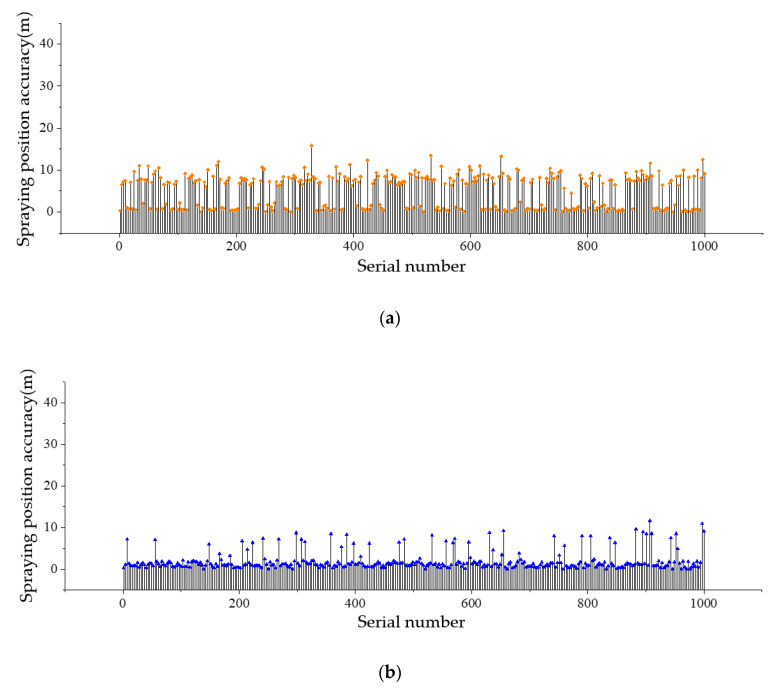

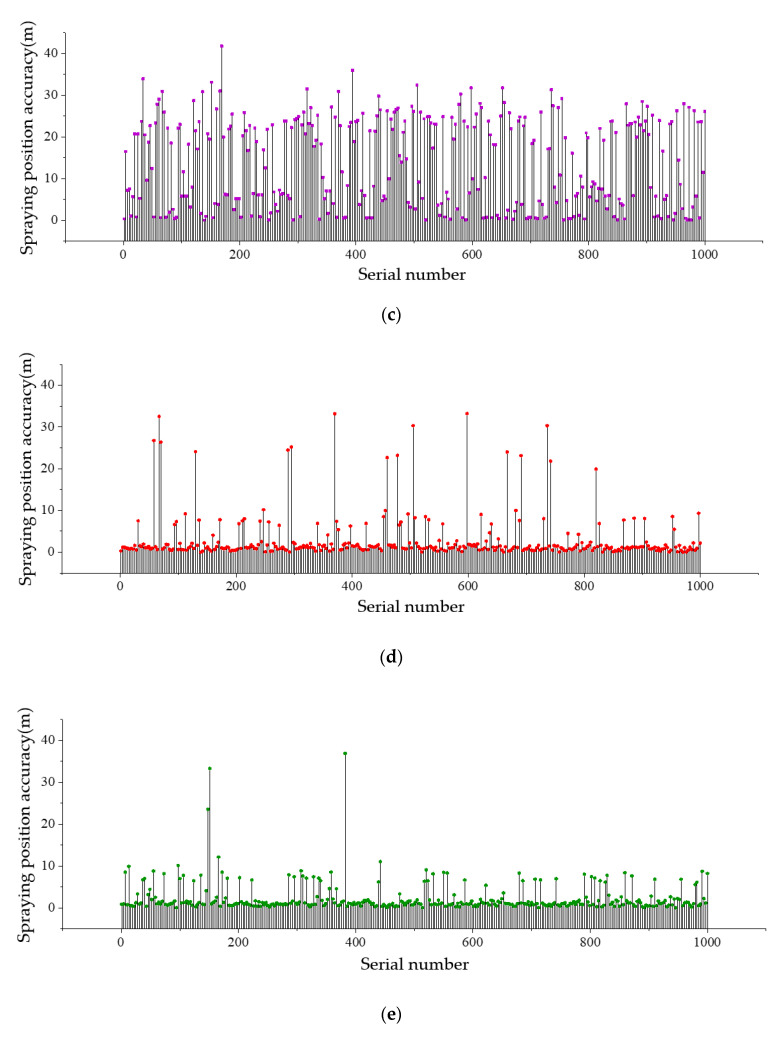

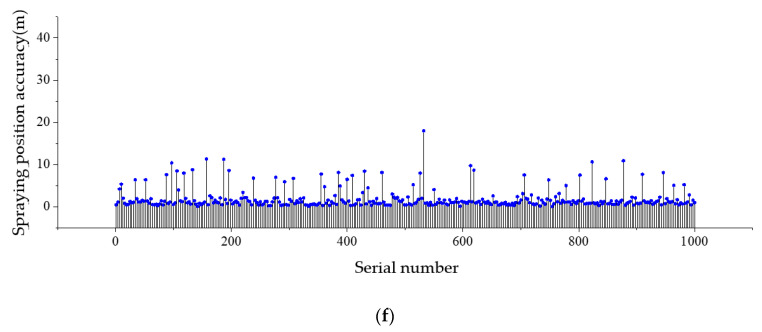
Spraying accuracy of PSO optimization under different wind data. (**a**) no route adjustment; (**b**) route adjustment with current wind data; (**c**) route adjustment with predicted wind data; (**d**) route adjustment with combined wind data (W = 0.35 m/s); (**e**) route adjustment with combined wind data (W = 0.5 m/s); (**f**) route adjustment with combined wind data (W = 1 m/s).

Different types of wind speed and direction data are applied to PSO-based trajectory optimization, as shown in Figure 13a–f. It can be seen from Figure 13a that the pesticide spraying effect is poor without trajectory adjustment. In Figure 13b, using the currently received wind speed and direction data for trajectory optimization for the next 10 s can significantly improve the spraying accuracy. As can be observed from Figure 13c, the spraying accuracy of new route optimized with the predicted wind speed and direction data is even worse than that without trajectory adjustment. Nonetheless, data distribution is polarized in observation. In Figure 13d–f, the predicted wind speed and direction data are used for trajectory optimization when the average wind speed change in the past 10 s is greater than the set threshold, otherwise, the current wind speed and direction data will be used for the next ten-second trajectory optimization. Evidently, as the threshold value increases, the test group with a larger f value begins to disappear. When the threshold value is 1 m/s, the performance effect is comparable to that in Figure 13b.

The average spraying accuracy by using different wind speed and direction data in the DQN and PSO trajectory optimization is presented in Table 5. The average spraying accuracy $\bar{f}$ is obtained upon the test of 1000 sets of data. It can be seen that trajectory optimization using the combined wind data of the current and the predicted with the threshold of 1 m/s present a slightly improved effect, in comparison to the trajectory optimization using merely the current wind direction and speed data. It is because predicting the wind data of the next time period based on the recent changes in wind speed and direction can capture some changing trend of wind in the case of great changes happening, which is more effective than merely using the current wind speed and direction to replace the future wind speed and direction.

## 4. Hardware Testing and Implementation

In this section, the communication time between UAV and WSN is tested, as well as the feasibility of running the PSO-based trajectory adjustment on the embedded hardware is verified.

### 4.1. Communication Test between UAV and WSN

**Figure 14:**
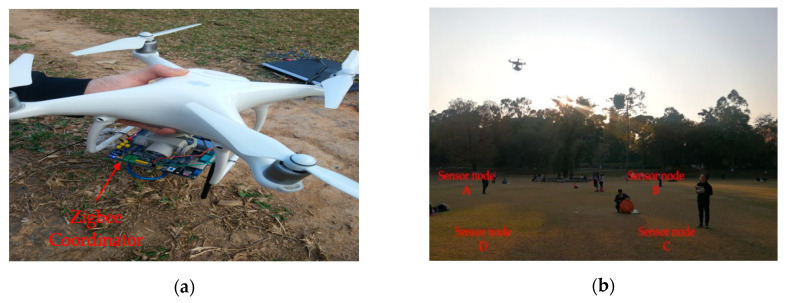
Communication test between UAV and ground WSN nodes. (**a**) UAV with Zigbee Coordinator; (**b**) Ground wireless sensor nodes.

CC2630 Zigbee modules were used in this test for networking, one of which was placed on the DJI Drone Phantom IV and configured as a coordinator, as shown in Figure 14a, while the other four modules were applied for simulating ground sensor nodes, and configured as terminals nodes, as shown in Figure 14b.

**Figure 15:**
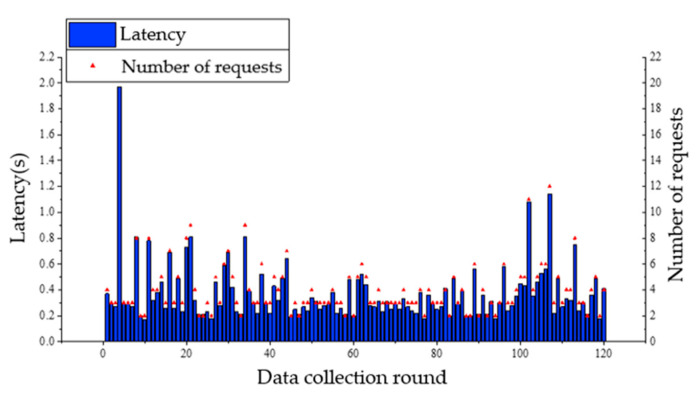
Communication latency of node A and the number of requests from UAV when flight altitude is 5 m.

**Figure 16:**
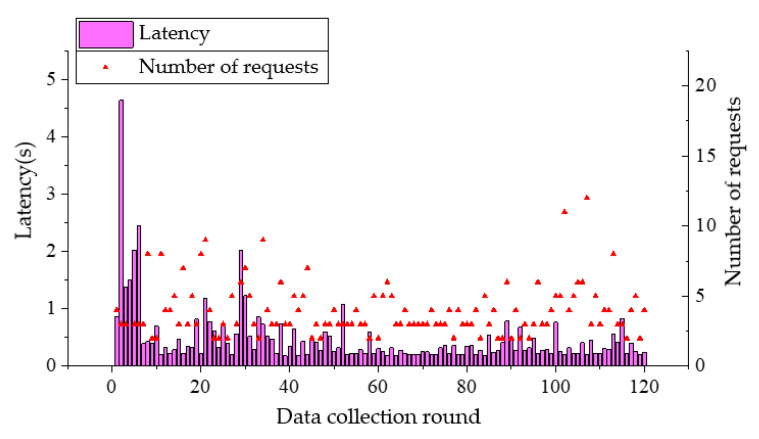
Communication latency of node A and the number of requests from UAV when flight altitude is 10 m.

**Figure 17:**
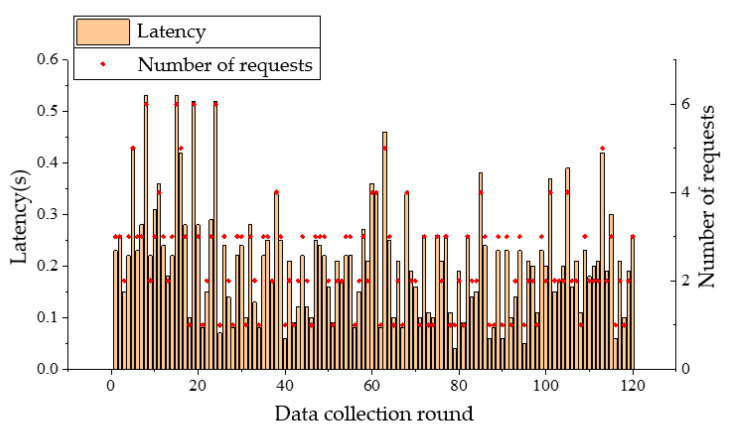
Communication latency of node B and the number of requests from UAV when flight altitude is 5 m.

**Figure 18:**
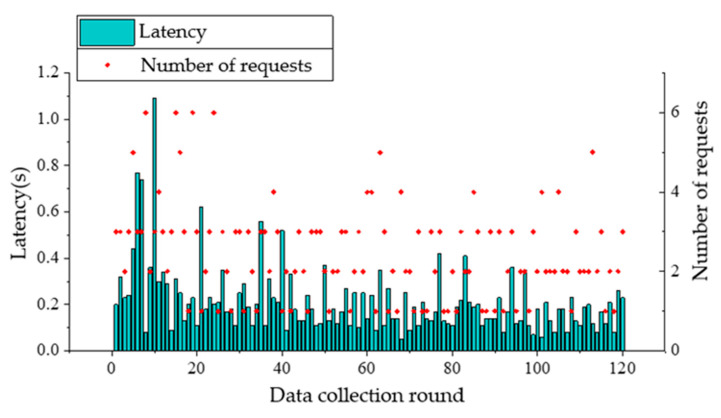
Communication latency of node B and the number of requests from UAV when flight altitude is 10 m.

During the experiments, it was found that without obvious obstacles, the received signal became weak and the communication was affected when the plane projection distance between the UAV and the nearest node on the ground exceeds 80 m. In the following tests, ground nodes are arranged at the four corners of a rectangle with 50 m in length and 30 m in width. When the UAV flies at a speed of 5 m/s with the respective altitude of 5 m and 10 m, it starts from the midair above node A, and flies to node B, node C, and node D in turn, and finally returns to node A without hovering. The UAV sends a data request to the ground nodes during flighty. A retransmission mechanism is adopted in the data transmission of nodes. And the UAV will send a new data request upon receiving data from all nodes. A total of 120 rounds of data collection are carried out in a single flight. Communication latency of nodes A and B and the number of requests sent by the coordinator to the node in each data collection are presented in Figure 15, Figure 16, Figure 17 and Figure 18. From the comparison between Figure 15 and Figure 16, and the comparison between Figure 17 and Figure 18, it can be seen that the UAV flight height of 5 or 10 m has little effect on the communication delay. Therefore, the impact of the plant protection UAV’s flight height on communication is ignored in this study when considering the deployment of wireless sensor nodes on the ground.

**Figure 19:**
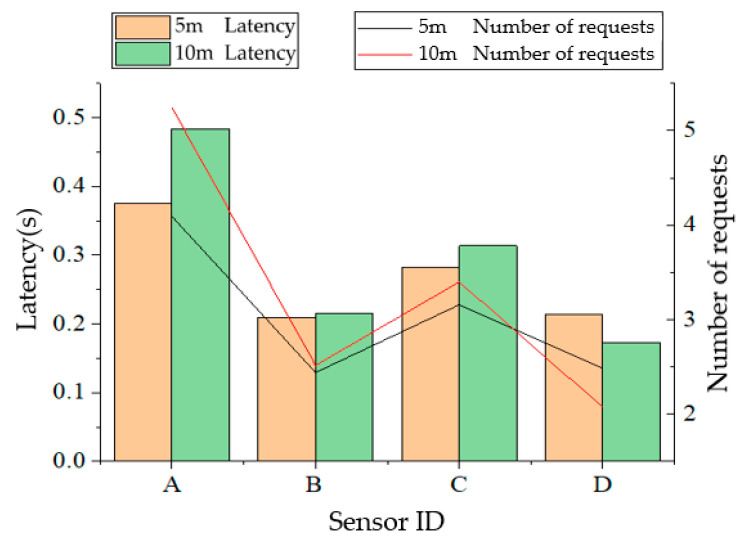
Average transmission latency of each node and average number of requests received.

The average communication latency between the UAV and the average number of requests to each node in each data collection is shown in Figure 19. As can be observed from Figure 19, the communication latency between each node and the UAV is less than 0.5 s for most of the rounds with few rounds greater than 1 s, showing a satisfactory network communication performance. In other words, latency can meet the real-time requirement of the UAV trajectory adjustment. By observing the average transmission latency of each node and the average number of requests received, it can be found that the average latency of node A is less than 0.5 s, and the average latency of other nodes is between 0.15 s and 0.3 s. Hence, speculation that some inference might present in the vicinity of node A can be made. At the same time, the average communication latency difference of each node when the UAV flight altitude is 5 m and 10 m is less than 0.1 s, indicating that the flight altitude has little effect on the communication latency in low-altitude flight.

### 4.2. Realization of the Scheme on Embedded Hardware

**Figure 20:**
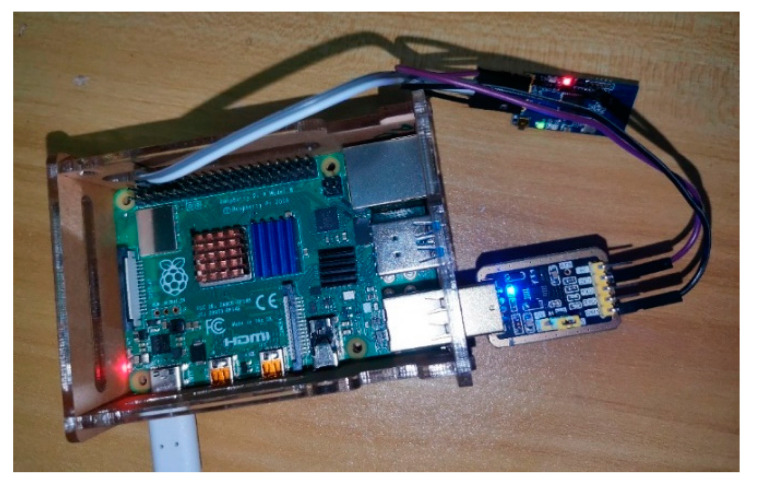
Algorithm evaluation on Raspberry Pi 4B+.

The wind speed and direction prediction model and the PSO-based trajectory adjustment algorithm are operated on the Raspberry Pi 4B+ (as shown in Figure 20), so as to evaluate whether they can meet the real-time requirements of trajectory adjustment of UAV. Because Python and TensorFlow have good portability and version compatibility, the PSO path optimization simulation program, the wind speed and direction prediction model, and the library files used for simulation testing on the embedded hardware device-Raspberry Pi 4B+ remain the same as those on the PC. Due to the limitations of the Raspberry Pi GPU performance and related code library support, the Raspberry Pi uses the CPU processor to infer the wind speed and direction prediction model, which is different from the PC end.

**Figure 21:**
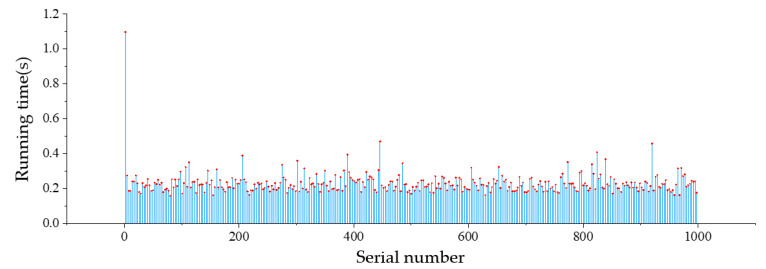
Running time of the wind prediction model and PSO-based algorithm on Raspberry Pi 4B+.

3000 sets of data are randomly selected from the collected wind speed and direction data set. 10 wind speed and direction values in consecutive time are included in each set of data, and the time corresponding to each set of wind speed and direction values is a cycle of trajectory adjustment. The running time of the test is stipulated as follows: It starts by receiving current wind data, then predicting wind speed and direction, and it is not stopped until parameters of PSO-based trajectory adjustment is output. The running time for obtaining parameters of trajectory adjustment of the first 1000 sets of data is shown in Figure 21. The possible reason for the long running time of the first set of data is that the processor needs to load the corresponding library file for the first time. The average running time of 3000 sets of data is 0.229 s. That is to say, the average time consumed by the Raspberry Pi from receiving environmental data to outputting trajectory fine-tuning parameters is 0.229 s. Considering that the communication latency between the UAV and the ground nodes is about 0.5 s, the communication time and the algorithm running time test show that the WSN-based trajectory adjustment system of UAV in this study can meet the real-time requirements in the actual pesticide spraying operation.

## 5. Conclusions

A system was designed for adjusting the spraying trajectory of a crop protection UAV based on data obtained from ground wireless sensors with the view to controlling pesticide drift. First of all, a UAV droplet drift model under the influence of wind speed and direction was established. On this basis, the UAV spraying trajectory was optimized using DQN and PSO algorithms, respectively. The simulation results show that both algorithms can decrease the droplet drift dramatically with the reduction in the drift distance of the droplet deposition center by up to 50%.

Moreover, LSTM and RNN were applied for predicting wind speed and wind direction, respectively, so as to address the problem of missing the latest wind speed and direction data caused by communication latency or lack of connection with the ground nodes. The simulation results show that using the predicted wind speed and direction value for trajectory optimization can further enhance the pesticide drift control effect in comparison to using merely the current wind speed and direction value for trajectory optimization, when the average wind speed change is greater than 1 m/s in a short period of time in the past. 

At last, the implementation of the UAV route adjustment system was discussed. The communication test between the UAV and the ground WSN proves that the communication latency is, in general, within 0.5 s. Also, the average time consumed for running the wind speed and direction prediction model and the PSO-based trajectory optimization algorithm on the Raspberry Pi 4B+ is 0.229 s, indicating that the UAV trajectory adjustment system can meet the real-time requirement in the pesticide spraying operation.

Researches concerning the following aspects will be carried out. Firstly, since the droplet drift model used in this study is only for one UAV model, the droplet drift and deposition in more mainstream UAV models and nozzles under the influence of wind speed and direction should be studied in the future. Moreover, relevant research on the relationship between the deposit amounts on the leaf wetness sensor and the deposition time would be carried out, in order to provide the operation guiding for UAV trajectory adjustment or support the analysis of deposition indicators upon spraying. In addition, the accuracy of wind direction and wind speed sensors also affect the accuracy of trajectory adjustment. The wind direction sensors used in this study can only acquire wind directions in eight orientations. In order to reduce the error, sensors with higher accuracy are required. Finally, the flight control system of the UAV will be developed, so that the trajectory adjustment method can be verified in the field test.

## Figures and Tables

**Table 1:** Wind speed and direction data for the algorithms.

Wind Speed (m/s)	Wind Direction (Radian)
0.9	π/2
2.1	π/2
3.5	3π/4
2.1	3π/4
2.7	3π/4
2.7	π/2
4.9	π/2
7.5	π/4
6.5	π/4
5.5	π/4

**Table 2:** DQN training parameters.

Parameters	Value
Learning rate α	0.001
Discount factor γ	0.95
Exploration rate $\varepsilon^{t}$	0.99$\varepsilon^{t\;-1}$
Replay memory size	128,000
Network training times	300

**Table 3:** PSO parameter settings.

Parameters	Value
Number of particles	20
Max iterations	100
Learning factors c1, c2	1.4961

**Table 4:** Changes in $∆v_{f\;},\;∆\theta_{f}\;,{\;t}_{0\;}\;$ and *f* ($∆v_{f\;},\;∆\theta_{f}\;,{\;t}_{0\;}$) in the iterative process.

$∆v_{f\;}(m/s)$	$∆\theta_{f}(\mathrm{Radian})$	$\;t_{0\;}(s)$	$f(∆v_{f\;},\;∆\theta_{f}\;,\;t_{0\;})$
−0.4	−π/18	2	5.544
−0.2	−π/36	3	5.326
−0.2	−π/36	3	5.326
−0.2	−π/30	2	5.263
−0.2	−π/30	2	5.263
−0.3	−π/45	2	5.123
−0.2	−π/60	4	4.963
−0.1	−π/180	7	4.937
−0.1	−π/90	7	4.750
−0.4	−π/18	2	5.544
−0.2	−π/36	3	5.326

**Table 5:** Average spraying accuracy under different wind data.

Settings	Metaheuristic	Average Spraying Accuracy(m)
No RA	None	5.34736
RA with current wind data	DQN	1.62043
RA with predicted wind data	DQN	10.48642
RA with combined wind data (W = 0.34 m/s)	DQN	2.33512
RA with combined wind data (W = 0.5 m/s)	DQN	1.98373
RA with combined wind data (W = 1 m/s)	DQN	1.47751
RA with current wind data	PSO	2.03988
RA with predicted wind data	PSO	13.99703
RA with combined wind data (W = 0.34 m/s)	PSO	2.99026
RA with combined wind data (W = 0.5 m/s)	PSO	2.3516
RA with combined wind data (W = 1 m/s)	PSO	1.96046
